# Formulation of Neem and Echinacea Gel for Oral Health Along With the Evaluation of Antimicrobial, Cytotoxic, Anti-inflammatory, and Free Radical Scavenging Activity: An In Vitro Study

**DOI:** 10.7759/cureus.63631

**Published:** 2024-07-01

**Authors:** Vyshnavi B Sindhusha, Arvina Rajasekar

**Affiliations:** 1 Periodontics, Saveetha Dental College and Hospitals, Saveetha Institute of Medical and Technical Sciences, Saveetha University, Chennai, IND

**Keywords:** azadirachta indica (neem extract), antimicrobial activity in natural products, generalized periodontitis, local drug delivery agents, echinacea

## Abstract

Background

Herbs have been used in medical practice for centuries and continue to play a significant role in modern complementary and alternative medicine. Phytochemicals in these herbs possess strong antioxidant and anti-inflammatory properties, which are beneficial in targeting oral health issues, such as dental plaque, gingivitis, and oral microbial infections. As research progresses, the challenge remains to translate these natural compounds into safe, effective, and accessible treatments for a wide range of diseases.

Aim

The aim of this research was to formulate the neem and echinacea gel along with the evaluation of antimicrobial, anti-inflammatory, free-radical scavenging activity, and cytotoxic potential.

Materials and methods

The neem and echinacea gel was prepared using a concentrated powdered mixture of neem and echinacea (5 grams each) to which 100 ml of distilled water was added, and the mixture was boiled for 30 minutes at 60°C. The 10 ml concentrate was mixed with 20 ml of a carbopol and carboxymethyl cellulose (CMC) mixture and mixed thoroughly, which resulted in neem and echinacea gel. Then, the antimicrobial, anti-inflammatory, cytotoxic potential, and free-radical scavenging activity of the gel were evaluated. The data obtained were statistically analyzed with the help of a paired t-test, where a p-value of less than 0.05 was considered statistically significant.

Results

The antimicrobial assay showed that neem and echinacea gel at the concentration of 100 micrograms showed a greater zone of inhibition against S*taphylococcus aureus *(3.15 ± 0.26), S*treptococcus mutans *(2.48 ± 0.45), E*nterococcus faecalis *(2.89 ± 0.15), and *Candida albicans *(4.28 ± 0.87). The cytotoxic test revealed that even at an 80 µg concentration of the extract, more than 70% of the nauplii were vital, which indicated that the gel was not cytotoxic. The highest anti-inflammatory activity (78.39 ± 1.82) of the gel was seen at 50 micrograms when compared with diclofenac sodium (73.16 ± 1.80). The free radical scavenging activity showed that the 2,2-diphenyl-1-picrylhydrazyl (DPPH) absorbance of the neem and echinacea extract was highest at 50 micrograms.

Conclusion

The combination of neem and echinacea extract-based gel possessed high antimicrobial and anti-inflammatory activity when compared with standard drugs, such as amoxicillin and diclofenac sodium. The antioxidant activity of the gel was equal to butylated hydroxytoluene (BHT), and also the gel has a low cytotoxic potential even at its higher concentrations. Hence, the gel can be used as a natural remedy with minimal side effects, making it a valuable alternative to chemical agents.

## Introduction

Oral diseases are a significant health concern as they often result in pain and discomfort during chewing, leading to dietary restrictions and negatively impacting a patient's self-esteem and social interactions [1]. Moreover, the bacteria associated with oral diseases cause inflammation, which can contribute to the development of systemic diseases, such as cardiovascular diseases, diabetes, and respiratory infections [2]. Microorganisms contribute to various oral infections with the help of their virulence factors, such as exoproteins (nucleases, hemolysins, lipases, and proteases) that degrade nucleic acids, proteins, red blood cells, and fatty acids, resulting in tissue damage, destruction of host cell membranes, and nutrient acquisition [3]. Enterotoxins of the bacteria overstimulate the immune system and act as superantigens. Effective prevention and management strategies are essential in reducing the prevalence and impact of these diseases.

Various medicinal plants were used for healing purposes in various cultures, which include plant extracts, poultices, and infusions, as these plants possess anti-inflammatory, chemo-protective, and antioxidant activities [4]. Understanding the mechanism of action of herbal medicines helps in exploring the bioactive pathways in the plant to produce optimized therapeutic effects. The integration of traditional knowledge with modern scientific methods allows for a more comprehensive understanding of the therapeutic potential of medicinal plants and spices [5]. This approach contributes to the development of evidence-based practices in herbal medicine, ensuring that these remedies are both culturally grounded and scientifically validated for optimal patient care [6].

Echinacea (*Echinacea purpurea*) is a genus of herbaceous plants of North America. This plant is often studied for its potential immunostimulant properties along with its anti-inflammatory and antioxidant properties. It is a herbal ingredient used as a natural remedy for health and wellness [7]. Neem (*Azadirachta indica*) has a profound history of medicinal use. It contains a wide range of chemical constituents along with biologically active compounds, which possess immunomodulatory effects that contribute in enhancing the body's immune response [8]. In this context, the aim of the research was to formulate the neem and echinacea gel along with the evaluation of antimicrobial, anti-inflammatory, free-radical scavenging activity, and cytotoxic potential of neem and echinacea gel.

## Materials and methods

Formulation of echinacea and neem gel

**Figure 1:**
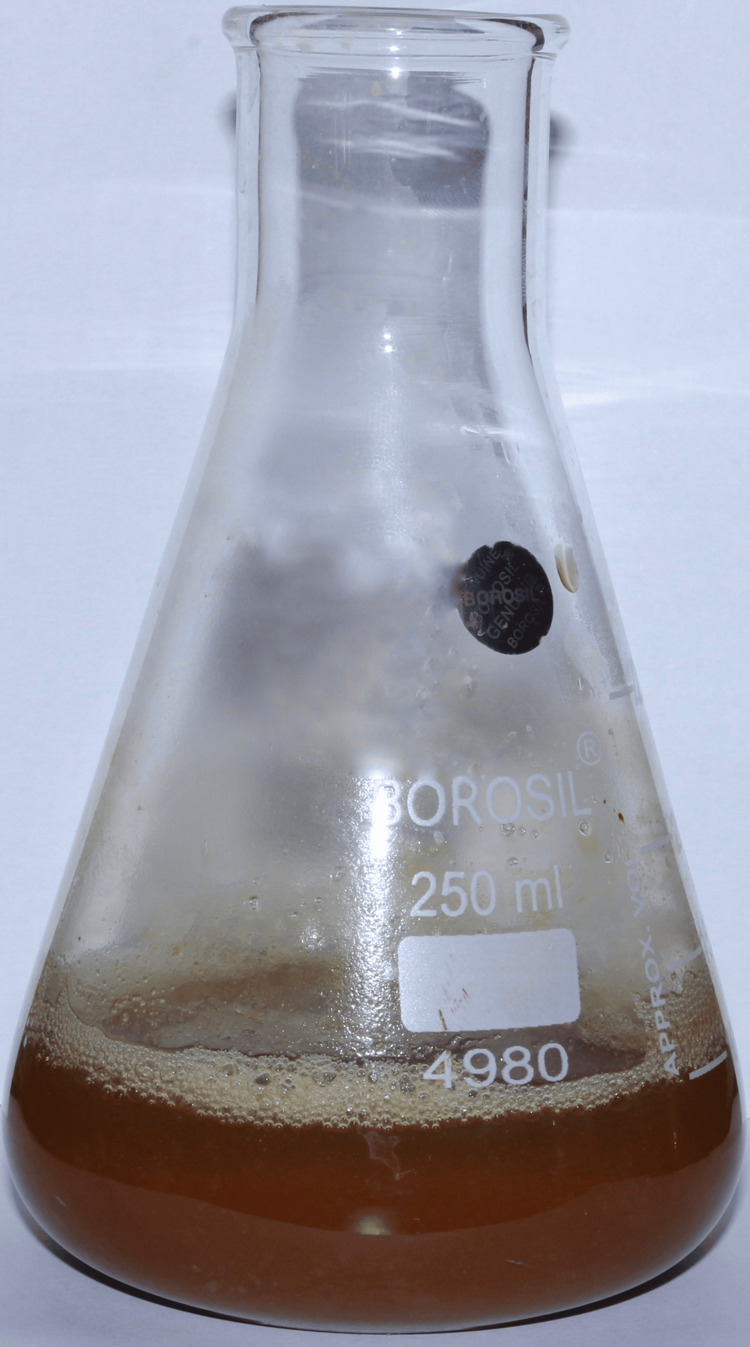
Neem and echinacea extract The above image depicts the thickened crude extract of neem and echinacea after boiling for 30 minutes at 60°C.

An in vitro analysis was performed after obtaining approval from the Institutional Scientific Review Board (SRB/SDC/PERIO-2104/24/090). Powdered neem and echinacea (5 grams each) were mixed in 100 ml of distilled water. Then, the mixture was boiled for 30 minutes at 60°C. The thickened and concentrated mixture was removed from the heat and cooled down from which the desired concentration (10 ml) was utilized for further use (Figure 1).

**Figure 2:**
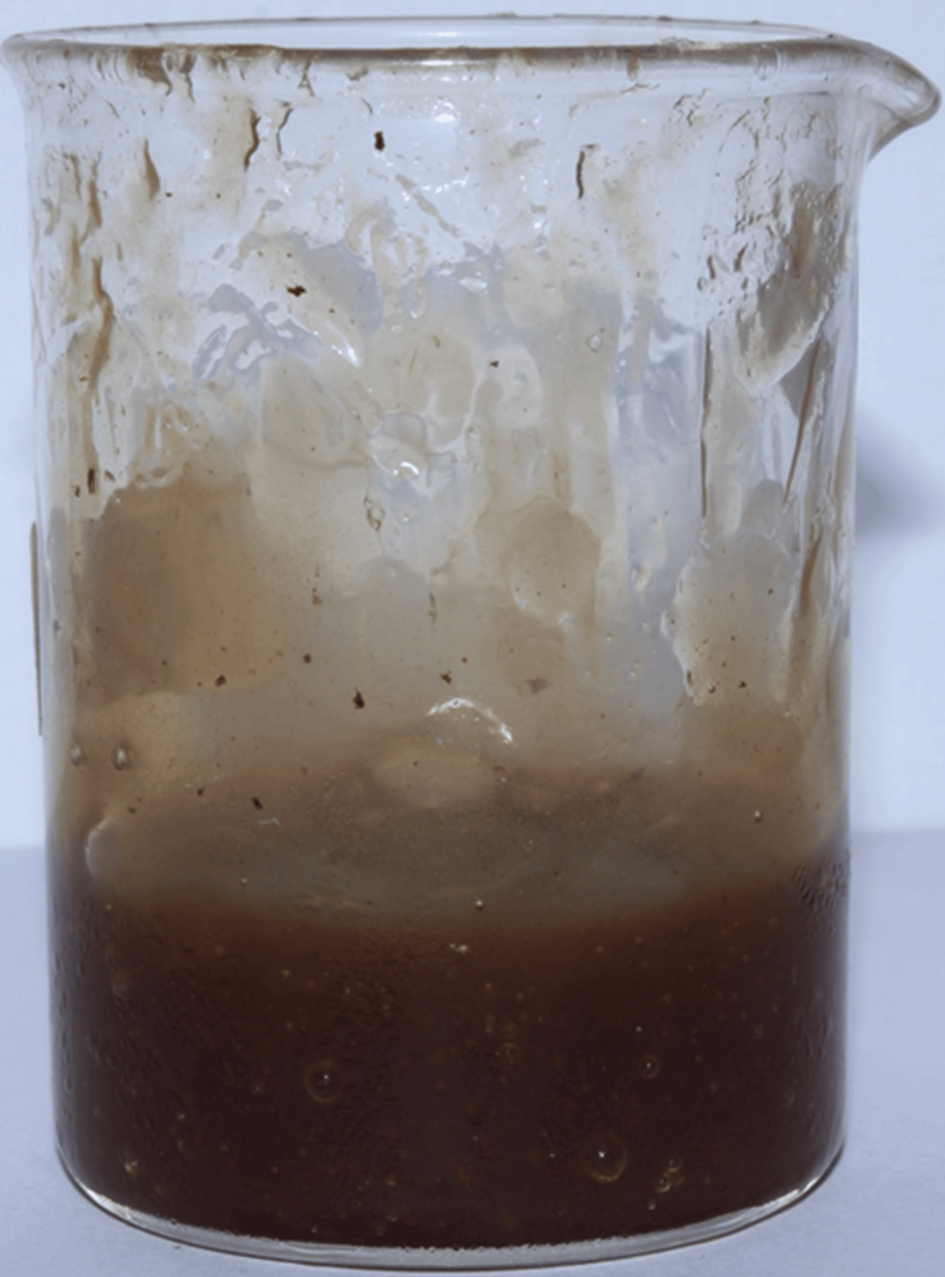
Neem and echinacea gel The 10 ml concentrate of neem and echinacea extract was mixed with the carbopol and carboxymethyl cellulose (CMC) mixture to obtain neem and echinacea gel.

Then, the 10 ml of the concentrated mixture of neem and echinacea was mixed with 20 ml of a carbopol and carboxymethyl cellulose (CMC) mixture, which was prepared by mixing 3 grams of each component in 20 ml of distilled water and well-dispersed. The contents were mixed thoroughly to ensure an even distribution of the neem and echinacea concentrate, and this resulted in a proper gel formation (Figure 2).

Antimicrobial activity

The antibacterial activity was assessed against *Staphylococcus aureus, Enterococcus faecalis, *and* Streptococcus mutans* using the agar well diffusion method [9]. The bacterial cultures were streaked on Mueller-Hinton Agar plates. Different concentrations (25 µg, 50 µg, and 100 µg) of the neem and echinacea gel were incorporated into the prepared wells, and the plates were incubated at 37°C for 24 hours to study its effect on the cultured bacteria. Amoxicillin was used as a positive control against bacteria. The zones of inhibition were recorded. For *Candida albicans*, rose bengal agar plates were used, and the incubation period was 24-48 hours. Fluconazole was used as positive control, and their zones of inhibition were recorded.

Cytotoxic analysis

Eggs of brine shrimp were incubated in artificial seawater that was made using 40 g/L of sea salt, 6 mg/L of dry yeast, and oxygenated using an aquarium pump [10]. Nauplii were harvested using a Pasteur pipette during a 48-hour incubation period in a warm room (between 22°C and 29°C). The organisms were initially drawn to one side of the tank using a light source. In tiny beakers filled with seawater, nauplii were pipetted two or three times in order to remove them from the eggs. Each well included 10 nauplii and varied concentrations (5 μg, 10 μg, 20 µg, 40 µg, and 80 µg) of neem and echinacea gel. For comparison, a control consisting solely of a NaCl solution and nauplii was also utilized. To determine the gel's lethality, the wells were left undisturbed for a whole day and the number of nauplii in each well was counted.

Anti-inflammatory activity

The anti-inflammatory activity of the neem and echinacea gel was measured using the protein denaturation activity of bovine serum albumin (BSA) [11]. Varied concentrations (10-50 μg) of the gel were added into five different test tubes along with the 2 ml of 1% BSA after which the pH of the reaction mixture was adjusted to 6.8 using 1N HCl. Then, the test tubes were incubated in a water bath at room temperature for 20 minutes, followed by measuring the absorbance of the reaction mixture at 660 nm using a spectrophotometer. Diclofenac sodium was used as the standard control.

Free-radical scavenging activity

The 2,2-diphenyl-1-picrylhydrazyl (DPPH) assay was used to evaluate the free radical scavenging activity of neem and echinacea gel [12]. Different concentrations (10 μg, 20 μg, 30 μg, 40 μg, and 50 μg) of the herbal gel was mixed with 1 ml of 0.1 mM DPPH in methanol solution and 450 μL of 50 mM Tris-HCl buffer (pH 7.4) and incubated for 30 minutes. After incubation, the reduction in the number of DPPH free radicals was measured using a spectrophotometer at 517 nm. Butylated hydroxytoluene (BHT) was used as a control. 

Statistical analysis

The data obtained were evaluated using IBM SPSS Statistics, version 23.0 (released 2014, IBM Corp., Armonk, NY). The antimicrobial, anti-inflammatory, and free radical scavenging activities were statistically analyzed with the help of paired t-tests, where a p-value of less than 0.05 indicates that the difference between the groups was statistically significant.

## Results

Antimicrobial activity

**Figure 3:**
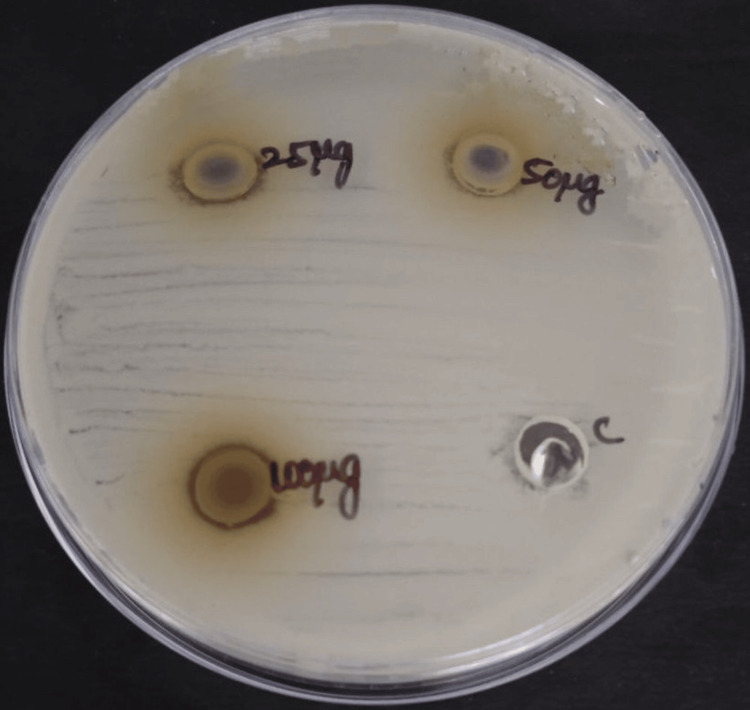
Antimicrobial activity of neem and echinacea gel against Staphylococcus aureus The antimicrobial activity was seen against *Staphylococcus aureus* in the agar medium with different concentrations of neem and echinacea gel (25 µg, 50 µg, and 100 µg) along with the control (amoxicillin).

**Figure 4:**
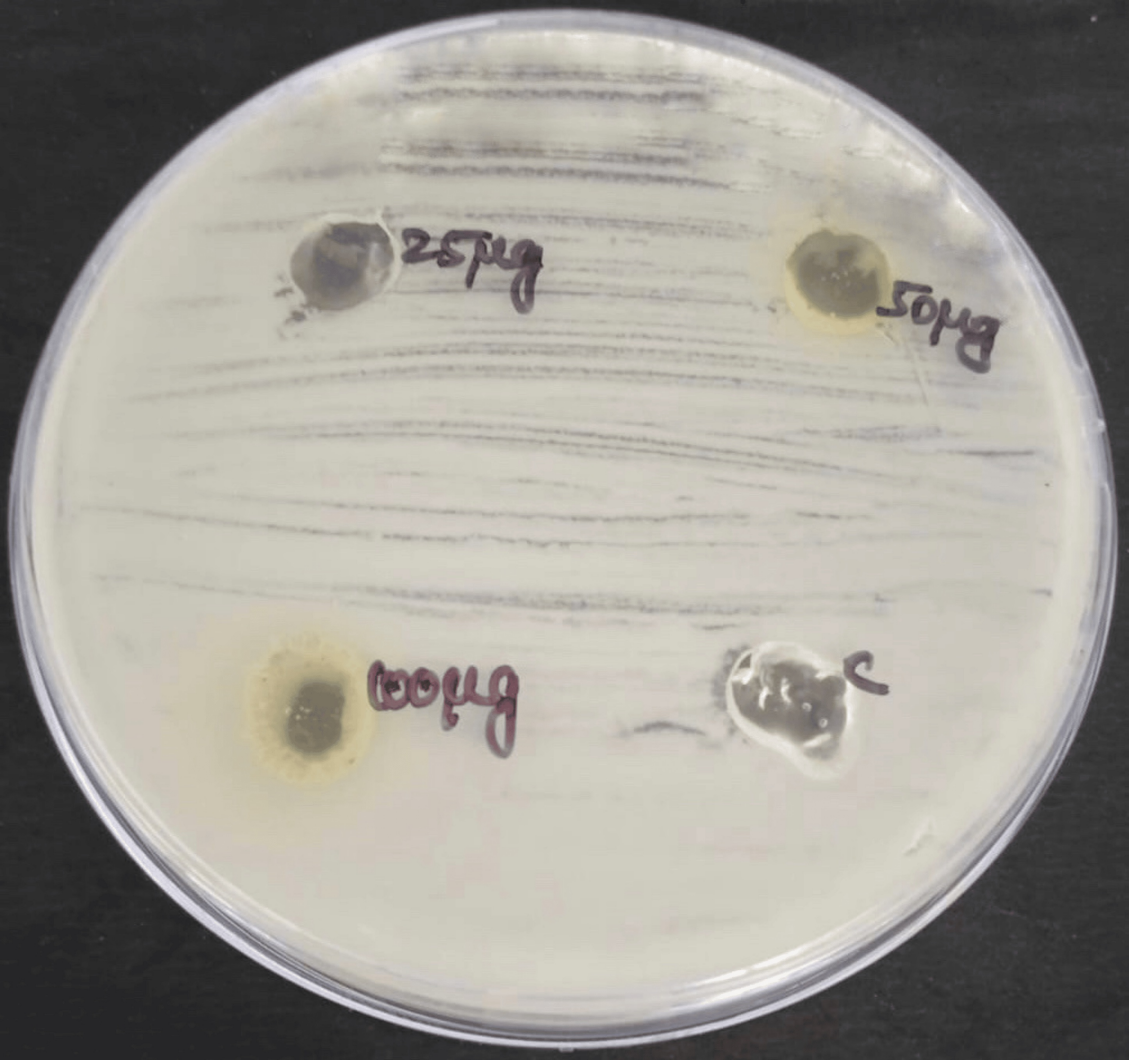
Antimicrobial activity of neem and echinacea gel against Enterococcus faecalis The antimicrobial activity was seen against* Enterococcus faecalis* in the agar medium with different concentrations of neem and echinacea gel (25 µg, 50 µg, and 100 µg) along with the control (amoxicillin).

**Figure 5:**
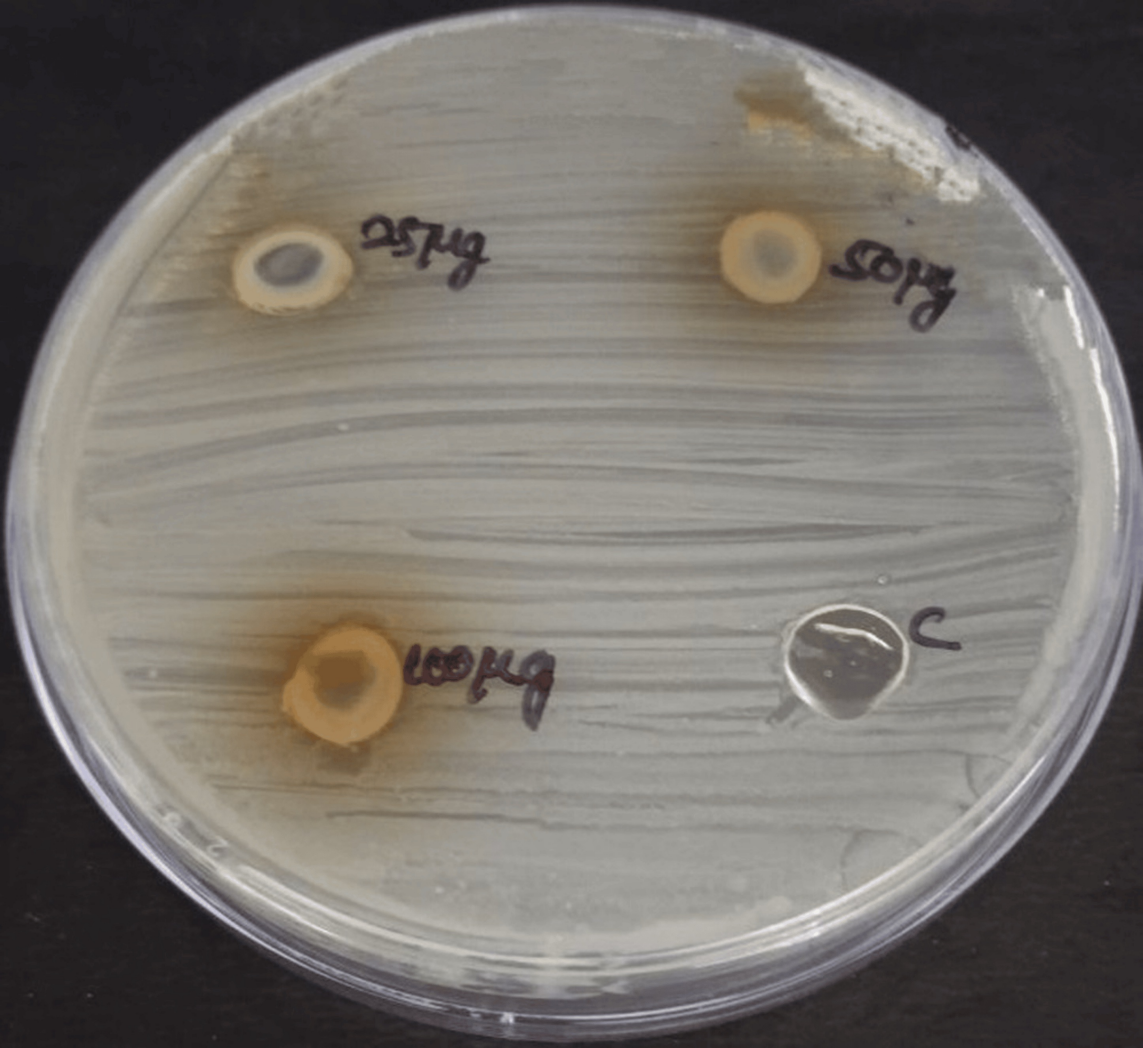
Antimicrobial activity of neem and echinacea gel against Streptococcus mutans The antimicrobial activity was seen against* Streptococcus mutans *in the agar medium with different concentrations of neem and echinacea gel (25 µg, 50 µg, and 100 µg) along with the control (amoxicillin).

**Figure 6:**
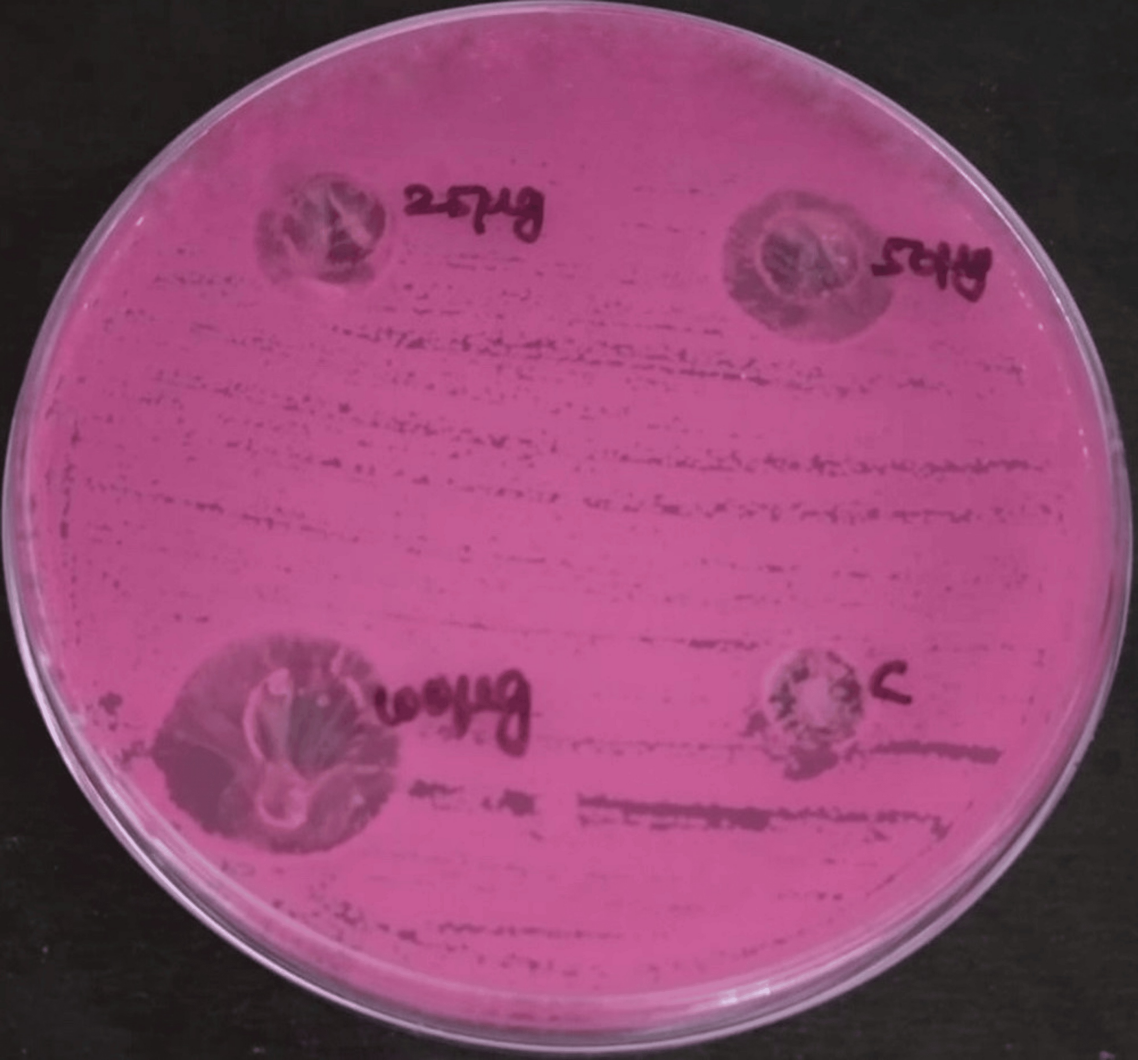
Antifungal activity of neem and echinacea gel against Candida albicans The antifungal activity was seen against *Candida albicans* with different concentrations of neem and echinacea gel (25 µg, 50 µg, and 100 µg) along with the control (fluconazole).

The antimicrobial activity of neem and echinacea gel was measured in terms of zone of inhibition against bacteria and fungi, such as *Staphylococcus aureus *(Figure 3*), Enterococcus faecalis *(Figure 4*), Streptococcus mutans *(Figure 5), and *Candida albicans *(Figure 6).

The mean zone of inhibition for various concentrations of neem and echinacea gel along with control is depicted in Table 1.

**Table 1:** The means and standard deviations of the zone of inhibition for different concentrations of neem and echinacea gel against different microorganisms (S. aureus, E. faecalis, S. mutans, and C. albicans)

Different concentrations of neem and echinacea gel	*Staphylococcus aureus* (zone of inhibition (mm) ± standard deviation (mm))	*Streptococcus mutans *(zone of inhibition (mm) ± standard deviation (mm))	*Enterococcus faecalis* (zone of inhibition (mm) ± standard deviation (mm))	*Candida albicans *(zone of Inhibition (mm) ± standard deviation (mm))
25 micrograms	0.18±0.04	0.23±0.06	0.16±0.13	1.45±0.26
50 micrograms	1.82±0.19	2.36±0.28	1.47±0.31	2.58±0.83
100 micrograms	3.15±0.26	2.48±0.45	2.89±0.15	4.28±0.87
Control	1.3±0.07	1.1±0.08	1.9±0.04	1.7±0.08

The study results showed that the highest zone of inhibition against all the microorganisms was observed at 100 micrograms of neem and echinacea gel when compared with the control. The zone of inhibition produced by neem and echinacea gel extract was higher when compared with the control, and the difference was statistically significant (p < 0.05).

Cytotoxic potential

The cytotoxicity of the neem and echinacea gel (5-80 µg) was evaluated by assessing the lethality of the nauplii against control. The lowest mortality of the nauplii was observed at 5 µg, and the highest was observed at 80 µg concentration of the herbal extract when compared to the control. Even at an 80 µg concentration of the extract, more than 70% of the nauplii were vital, which indicated that the neem and echinacea gel was not cytotoxic.

Anti-inflammatory activity

The results of the albumin denaturation assay are depicted as the mean and standard deviation in Table 2.

**Table 2:** The mean and standard deviation for the anti-inflammatory activity of neem and echinacea gel The anti-inflammatory activity of neem and echinacea gel was tested against diclofenac sodium. A paired t-test was used for the statistical analysis, and the P-value was found to be less than 0.05, which indicates that the difference was statistically significant.

Various concentrations (micrograms)	Neem and echinacea extract (mean ± standard deviation)	Diclofenac sodium (mean ± standard deviation)	P- value
10 micrograms	21.3±2.04	19.3±1.98	0.03
20 micrograms	29.09±2.58	26.87±3.20	0.05
30 micrograms	37.28±2.63	34.57±3.80	0.03
40 micrograms	52.47±3.42	50.16±1.78	0.04
50 micrograms	78.39±1.82	73.16±1.80	0.01

The neem and echinacea gel showed maximum anti-inflammatory activity of 78.39 ± 1.82 at 50 µg/ml when compared to diclofenac sodium (73.16 ± 1.80).

Free-radical scavenging activity

The results of the free-radical scavenging activity of herbal formulation and the control (butylated hydroxy toluene (BHT)) are depicted in Table 3.

**Table 3:** Free-radical scavenging activity of neem and echinacea gel The free-radical scavenging activity of neem and echinacea gel was tested against butylated hydroxy toluene (BHT). A paired t-test was used for the statistical analysis, and the P-value was found to be less than 0.05, which indicates that the difference was statistically significant. * The total antioxidant capacity was expressed as the mM equivalent of ascorbic acid per gram of dry weight.

Samples	2,2-Diphenyl-1-picrylhydrazyl (DPPH) IC50 values ± SE μg/ml	Total antioxidant capacity *
10 micrograms	4.15±0.23	0.32±0.36 mM
20 micrograms	5.16±0.09	0.42±0.58 mM
30 micrograms	7.12±0.03	0.58±0.09 mM
40 micrograms	7.28±0.05	0.69±0.03 mM
50 micrograms	8.23±0.46	0.72±0.05 mM
Control (BHT)	8.25±0.08	0.78±0.04 mM

The results revealed that the free-radical scavenging activity of neem and echinacea gel was the highest at 50 µg, followed by 40 µg, 30 µg, 20 µg, and 10 µg, when compared to the standard control.

## Discussion

Oral health conditions such as missing teeth or oral lesions can impair speech, affecting communication and social interactions along with restriction in dietary choices, impacting overall health. Poor oral health can lead to systemic conditions such as cardiovascular disease, diabetes, and adverse pregnancy outcomes [13]. The prevalence of oral diseases such as dental caries, periodontal diseases, and oral cancers is widespread and coupled with significant treatment costs, which underscores their status as a major public health problem [14]. The oral cavity provides a distinct habitat for various bacteria, fungi, and viruses where the host’s immune system interacts with this oral microbiota, maintaining a balance that prevents disease [15]. This balance between the oral microbial community and the host undergoes a constant shift due to changes in diet, oral hygiene, and other factors, leading to oral health issues [16].

The addition of antimicrobial agents systemically plays a crucial role in managing oral diseases [17]. However, these drugs cause various side effects such as kidney and liver toxicity, bone marrow suppression, and antimicrobial resistance [18]. The use of natural agents such as herbal extracts, essential oils, and plant-derived compounds has gained attention in recent years, as they have demonstrated antibacterial and anti-inflammatory properties along with the potential advantages, such as well tolerated and cost-effective. The formulation of herbs has the potential to target harmful microorganisms while preserving the natural flora of the oral cavity, and this elective action can help maintain a healthy equilibrium [19]. This approach is advantageous for individuals who seek alternatives to conventional treatment.

Neem (*Azadirachta indica*) has been widely known for its anti-inflammatory, antipyretic, and antihistamine properties as it contains bioactive compounds, such as nimbidol, sodium nimbinate, and azadirachtin, which contribute to a diverse therapeutic effect [20]. Echinacea contains alkamides, flavonoids, and polysaccharides, which enhance phagocytosis through the activation of T lymphocytes and the production of cytokines [21]. The previous literature has assessed the anti-inflammatory and therapeutic properties of neem and echinacea individually, where the concentration of 2 mg/ml of neem extract could inhibit 50% and 90% of bacterial strains tested across seven genera marking its broad-spectrum antimicrobial activity [22]. The present study is a first of its kind where the combination effects of neem and echinacea gel were evaluated for their antimicrobial, cytotoxic, anti-inflammatory, and free-radical scavenging activities. The results showed the inactivation of all the bacteria and fungi at 100 microgram concentration of gel when compared against amoxicillin and fluconazole, respectively. The cytotoxic test revealed that more than 70% of nauplii were alive even at an 80 microgram concentration, demonstrating that the neem and echinacea gel are not cytotoxic. Regarding the anti-inflammatory activity and the free-radical scavenging activity, the highest anti-inflammatory activity by the neem and echinacea gel was shown at 50 microgram concentration when compared against diclofenac sodium and the DPPH absorbance of the neem and echinacea gel was the highest at 50 micrograms, which is equal to the control (BHT). 

A study done by Altayb et al. [23] demonstrated that the methanolic extract of *A. indica* contains various bioactive compounds, such as beta-D-mannopyranoside and O-geranyl, which are capable of inhibiting the growth of various bacterial strains. Raghavendra et al. [24] studied the antifungal and antimicrobial activity of the neem, and the findings demonstrated superior results when compared with 3% sodium hypochlorite (NaOCl). The acetone extracts of neem were more effective at inhibiting bacterial growth compared to aqueous extracts, which indicates the influence of solvents against bacteria [25]. Neem chips have been used in addition to scaling, and root planing demonstrated better results in both clinical and microbiological parameters [26]. Moreover, a clinical trial suggested that neem chips showed benefits in the treatment of periodontitis [27]. 

Echinacea possesses antifungal and anti-inflammatory properties by inhibiting 5-lipoxygenase [28], and a pharmacological study has reported the anticancer and immunomodulatory effects of echinacea [29]. Bajrai et al. [27] screened the antiviral effects of echinacea using quantitative real-time polymerase chain reaction and demonstrated significant inhibition of the viral load. The microbicidal and immunomodulatory activity of echinacea was studied by Dosoky et al. [30], where echinacea showed a relatively high microbicidal activity against a variety of bacterial pathogens and has immunomodulatory effects on neutrophil activation. Our findings are in agreement with the previous findings. Therefore, these two herbs upon combining would offer a synergistic approach to combating bacterial infections and reducing inflammation. 

The limitations of the above-mentioned study are it is an in vitro study and is considered as a low level of evidence as they are conducted in artificial laboratory environments, which may not fully represent the complexities of the human body or natural conditions. Moreover, as in vitro experiments are short-term, they may not capture the long-term effects of interventions. Further in vivo studies and clinical trials should be done to validate these findings.

## Conclusions

The combination of neem and echinacea extract-based gel possesses high antimicrobial, anti-inflammatory, and antioxidant activities. Moreover, the gel was found to be non-cytotoxic even at high concentrations. Hence, this gel can be considered a promising substitute for conventional antibiotics in the management of oral diseases. Further research focusing on the in vivo effects of neem and echinacea is required to better understand its specific role. To fully comprehend its unique function, more studies concentrating on the in vivo effects of neem and echinacea are necessary.
